# Cleanliness

**Published:** 1859-03

**Authors:** 


### CLEANLINESS.

The virtue of cleanliness is characteristic of civilization. The higher the grade of their social advancement, the more apt are a people to rid themselves "of the earth earthy." Cleanliness, indeed, is the heraldry of genuine aristocracy. The man who is content to go dirty, is a born plebeian, whatever may be his wealth, talents, or family. Yes,

> "The man who hath no cleanness in himself,
> Nor is not moved toward washing with clean water,
> Is fit for gutters, still-slop-pens, and fleas:
> Let no such man be trusted."

And Shakspeare never uttered a truer sentiment. And it may here be remarked, incidently, that Mr. Shakspeare was generally about right in his observations of men.

The justness of the foregoing will be admitted at once; and yet who keeps his mouth clean?—who of all "the upper ten thousand?"—who of all these fine ladies and gentlemen?—who of all these "white sepulchers," who has not his mouth "full of dead men's bones and all uncleanness?" This beautiful lady, so immaculately clean, (in her own conceit,) has, at this moment, between her teeth great slivers of putrified and putrifying hog and cow, that have been lodged there since day before yesterday. All the aromatics of a wholesale apothecary shop would not, my dear Miss, avail to smother up the *rank sweetness of thy words:* thy very speech has a smack that the kissing winds prate of.

Clean your mouth! Not once a week; not once a day; but every time you have taken food. What would be thought of you if you did not wash your face as often as it became dirty? Or, would you expect to be tolerated in decent society, if you let your finger-nails grow so unclean as to rot off? Ought you, then to be tolerated there, when you treat your teeth in a similar manner? Think of it. Make a note of it. Be advised, for instance, as follows:

As soon as you are up in the morning, take water and a toothbrush, and thoroughly *scour* your mouth, (including your teeth, of course). Immediately after every eating—even of the least thing—clean the teeth carefully with a quill toothpick; then draw between them, where they stand close together, a thread of very fine, strong silk, so as to remove every substance that may have become wedged between them; and, finally, brush them vigorously with clean water. As Martin Farquhar Tupper would express it,

> Show me a man or a woman that keepeth the mouth clean,
> And I will show thee a person of good breeding.

